# A map of the PGC-1α- and NT-PGC-1α-regulated transcriptional network in brown adipose tissue

**DOI:** 10.1038/s41598-018-26244-4

**Published:** 2018-05-18

**Authors:** Ji Suk Chang, Sujoy Ghosh, Susan Newman, J. Michael Salbaum

**Affiliations:** 1 Laboratory of Gene Regulation and Metabolism, Pennington Biomedical Research Center, Baton Rouge, LA 70808 USA; 2 Genomics Core, Pennington Biomedical Research Center, Baton Rouge, LA 70808 USA

## Abstract

Transcriptional coactivator PGC-1α and its splice variant NT-PGC-1α play crucial roles in regulating cold-induced thermogenesis in brown adipose tissue (BAT). PGC-1α and NT-PGC-1α are highly induced by cold in BAT and subsequently bind to and coactivate many transcription factors to regulate expression of genes involved in mitochondrial biogenesis, fatty acid oxidation, respiration and thermogenesis. To identify the complete repertoire of PGC-1α and NT-PGC-1α target genes in BAT, we analyzed genome-wide DNA-binding and gene expression profiles. We find that PGC-1α-/NT-PGC-1α binding broadly associates with cold-mediated transcriptional activation. In addition to their known target genes in mitochondrial biogenesis and oxidative metabolism, PGC-1α and NT-PGC-1α additionally target a broad spectrum of genes involved in diverse biological pathways including ubiquitin-dependent protein catabolism, ribonucleoprotein complex biosynthesis, phospholipid biosynthesis, angiogenesis, glycogen metabolism, phosphorylation, and autophagy. Our findings expand the number of genes and biological pathways that may be regulated by PGC-1α and NT-PGC-1α and provide further insight into the transcriptional regulatory network in which PGC-1α and NT-PGC-1α coordinate a comprehensive transcriptional response in BAT in response to cold.

## Introduction

Brown adipose tissue (BAT) is specialized for heat production in rodents and humans^1,2^. Upon cold exposure, non-shivering thermogenesis in BAT is rapidly activated by the sympathetic nervous system that releases noradrenaline and concomitantly stimulates β-adrenergic receptors (AR) on BAT. This stimulation drives transcription of genes involved in mitochondrial biogenesis, respiration and thermogenesis. High levels of electron transport chain complexes create a fuel-derived proton electrochemical gradient across the mitochondrial inner membrane. A key thermogenic uncoupling protein 1 (UCP1) in turn decreases this proton gradient by promoting proton leak from the outer to the inner mitochondrial membrane, resulting in heat production instead of ATP synthesis^3,4^. Although mitochondrial respiration and UCP1-driven uncoupling are critical for maximal heat production in BAT, effective thermogenesis also relies on diverse biological processes, such as lipolysis^1,5^, fatty acid uptake and β-oxidation^6–8^, lipogenesis^9,10^, glucose uptake and metabolism^11,12^, etc. Genome-wide BAT transcriptome analyses have revealed that a number of genes associated with these processes are upregulated by cold/β-adrenergic stimulation of BAT^10,13–16^.

Transcriptional coactivator PGC-1α (797 aa), encoded by the *PPARGC1A* gene, is a key regulator of cold-induced BAT thermogenesis^17^. PGC-1α is highly induced by β-adrenergic stimulation and coordinates a transcription program resulting in increased mitochondrial biogenesis, fatty acid oxidation, respiration and thermogenesis by binding to and coactivating many different transcription factors, such as PPARs, ERRs, TRs and NRFs^17–19^. We previously reported a splice variant of the *PPARGC1A* gene that encodes a shorter isoform of PGC-1α (NT-PGC-1α, 270 aa) in rodents and humans^20^. NT-PGC-1α is a functional transcriptional coactivator retaining the transcriptional activation and nuclear receptor binding domains of full-length PGC-1α^20–24^. NT-PGC-1α is co-expressed with PGC-1α in BAT and is highly induced by cold^20,21^. Our previous microarray analysis showed that NT-PGC-1α significantly upregulates genes involved in fatty acid transport and β-oxidation, TCA cycle, electron transport system, glycolysis, lipogenesis, and PPARα activation pathways in PGC-1α^−/−^ brown adipocytes^23^. In agreement with *in vitro* gene expression data, NT-PGC-1α is sufficient to activate cold/β-AR-stimulated thermogenesis in BAT deficient in full-length PGC-1α^21^. In contrast, deficiency in both PGC-1α and NT-PGC-1α significantly impairs BAT thermogenic function^25^. These findings thus suggest that PGC-1α and NT-PGC-1α cooperate together to activate cold-induced thermogenesis in BAT. To map the transcriptional network mediated by PGC-1α and NT-PGC-1α in greater detail, we have now conducted chromatin immunoprecipitation-sequencing (ChIP-seq) in BAT from cold-exposed mice to assess genome-wide DNA-binding patterns of PGC-1α and NT-PGC-1α using a well-characterized PGC-1α antibody that recognizes both PGC-1α and NT-PGC-1α isoforms^21,22^. In parallel, we have also analyzed cold-responsive gene expression profiles in BAT. Our combined analyses of ChIP-seq and gene expression data sets reveal a more comprehensive repertoire of PGC-1α-/NT-PGC-1α target genes induced by cold/β-AR stimulation in BAT.

## Results

### Genome-wide identification and characterization of PGC-1α and NT-PGC-1α binding sites in brown adipose tissue

**Figure 1:**
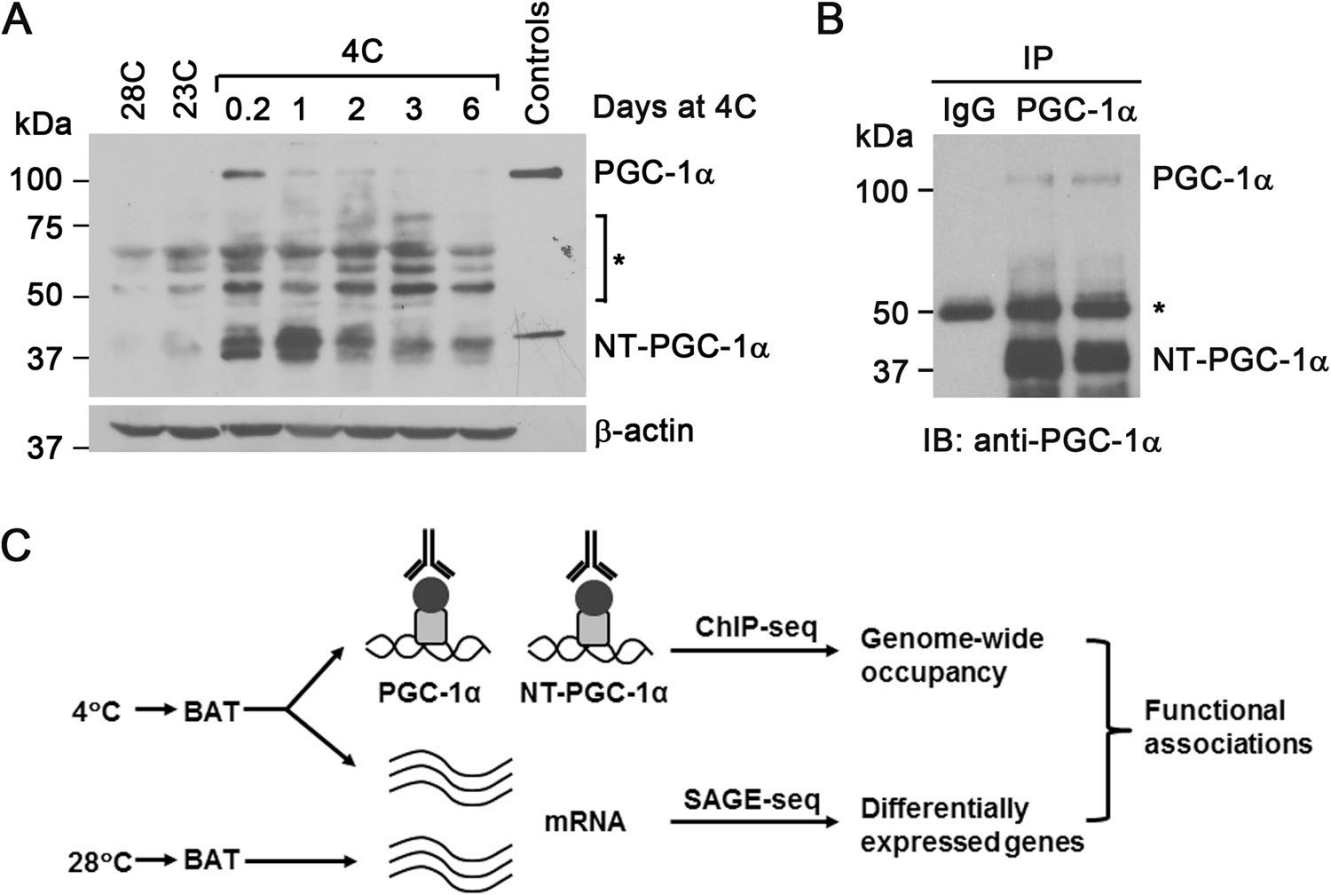
Cold-dependent increase of PGC-1α and NT-PGC-1α proteins in brown adipose tissue. (**A**) Expression of PGC-1α and NT-PGC-1α proteins in brown adipose tissue (BAT). BATs were isolated from mice housed at 23 °C, acclimated at 28 °C for 4 days, and exposed to 4 °C for 0.2, 1, 2, 3, and 6 days. HEK293 cells expressing PGC-1α-HA and NT-PGC-1α-HA were used as a positive control. *Represents non-specific bands. The cropped blots show PGC-1α, NT-PGC-1α, and β-actin. Full-length blots are presented in Supplementary Figure S1. (**B**) Validation of PGC-1α antibody for immunoprecipitation of PGC-1α and NT-PGC-1α. The protein lysates were prepared from BAT of mice exposed to 4 °C for 6 h. The cropped blot shows PGC-1α and NT-PGC-1α. *Represents the immunoglobulin heavy chain. A full-length blot is presented in Supplementary Figure S1. (**C**) Overall scheme to identify targets of PGC-1α and NT-PGC-1α in BAT and to investigate the cold-dependent regulation of PGC-1α and NT-PGC-1α target genes.

Transcriptional coactivators PGC-1α and NT-PGC-1α play a key role in transcriptional activation of cold-induced BAT thermogenesis^17,21,25^. To identify the complete repertoire of PGC-1α and NT-PGC-1α target genes in BAT, we performed chromatin immunoprecipitation with a previously validated PGC-1α antibody that recognizes both PGC-1α and NT-PGC-1α isoforms^21,22^, followed by high-throughput sequencing (ChIP-seq). Since mRNA and protein levels of PGC-1α and NT-PGC-1α are upregulated by cold in BAT^20^, we exposed C57BL/6 J mice to 4 °C for different amounts of time to find the condition where PGC-1α and NT-PGC-1α protein levels are maximal in BAT. PGC-1α and NT-PGC-1α were hardly detectable in BAT of mice housed at 28 °C and 23 °C, but their protein levels rapidly reached to a maximum at 6 h of cold exposure and decreased with longer exposure to cold (Fig. 1A). ChIP-seq was performed using 6h-cold exposed BAT samples where both PGC-1α and NT-PGC-1α protein levels are high. Given that both isoforms are immunoprecipitated by PGC-1α antibody (Fig. 1B), the binding profiles generated by ChIP-seq include targets of both PGC-1α and NT-PGC-1α (Fig. 1C). In parallel, to evaluate the functional association between genomic binding and gene expression, we conducted a genome-wide BAT transcriptome analysis using RNA isolated from BAT of mice housed at 28 °C or exposed to 4 °C for 6 h (Fig. 1C).

**Figure 2:**
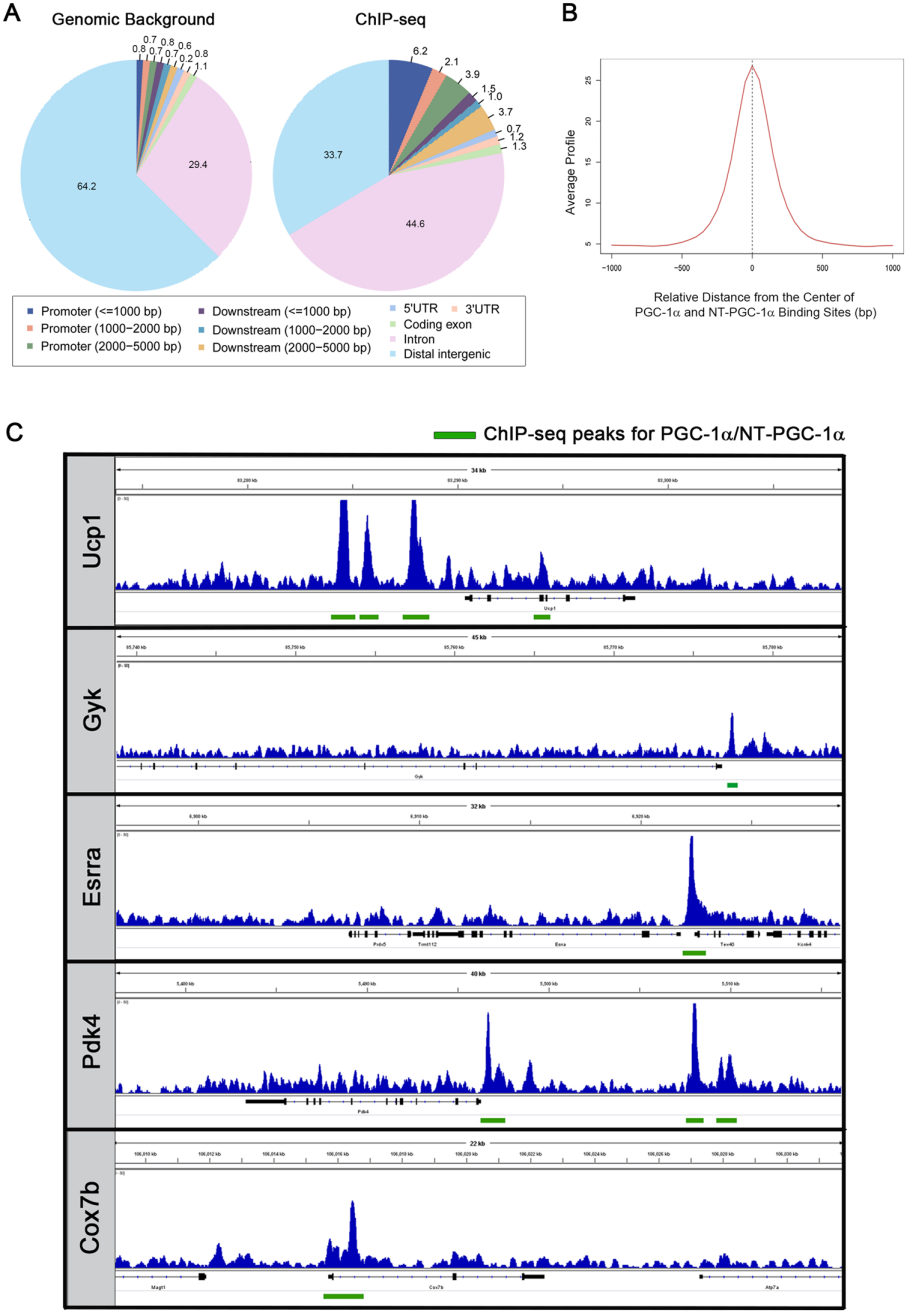
PGC-1α/NT-PGC-1α chromatin occupancy in brown adipose tissue. (**A**) Pie chart demonstrating the distribution of features in genome and among PGC-1α/NT-PGC-1α binding peaks. The listed genomic features include promoters (≤ 1 kb; 1 kb to 2 kb; 2 kb to 5 kb upstream of the transcription start sites); downstream elements (≤ 1 kb; 1 kb to 2 kb; 2 kb to 5 kb downstream of the transcription termination sites); gene body (5′ UTR, 3′ UTR, coding exons, and introns); and intergenic regions. (**B**) An average ChIP-seq signal profile around PGC-1α/NT-PGC-1α binding peaks. The average signal represents the average number of reads across the binding regions per 100 bp interval. (**C**) PGC-1α/NT-PGC-1α ChIP-seq binding profiles for known target genes (Ucp1, Gyk, Esrra, Pdk4, and Cox7b). Green boxes represent the binding site(s) for PGC-1α and NT-PGC-1α.

Peak calling by the Model-based Analysis of ChIP-Seq (MACS) algorithm identified 3,654 PGC-1α/NT-PGC-1α binding sites (FDR < 5%). Compared with the distribution of features in the mouse genome, PGC-1α/NT-PGC-1α binding sites were located within the 5 kb upstream of the transcription start sites (TSS) of the genes with 12.3% of all binding sites (Fig. 2A). Among the remaining sites, PGC-1α/NT-PGC-1α binding sites were located in ≤ 5 kb downstream of the transcription termination sites (6.2%), intragenic regions (44.6%), 5′- and 3′- UTR regions (1.9%), exons (1.3%), and intergenic regions (33.7%). The average peak signal at PGC-1α/NT-PGC-1α binding sites was highly enriched at the center of the binding regions (Fig. 2B). Binding events occurring at distal regions may suggest that PGC-1α and NT-PGC-1α regulate transcription of genes by binding to distal regulatory elements such as enhancers. In support of the validity of our ChIP-seq results, PGC-1α/NT-PGC-1α peaks were found adjacent to numerous previously identified target genes, including UCP1, GYK, ESRRA, PDK4 and COX7b (Fig. 2C)^17,26–28^. In case of UCP1 and PDK4, there were additional peaks located at the distal regions in addition to the peak at the promoter regions (≤5 kb of the TSS), suggesting that PGC-1α and NT-PGC-1α regulate gene expression at multiple sites at promoter and/or enhancer regions through cooperating with different transcription factors.

**Figure 3:**
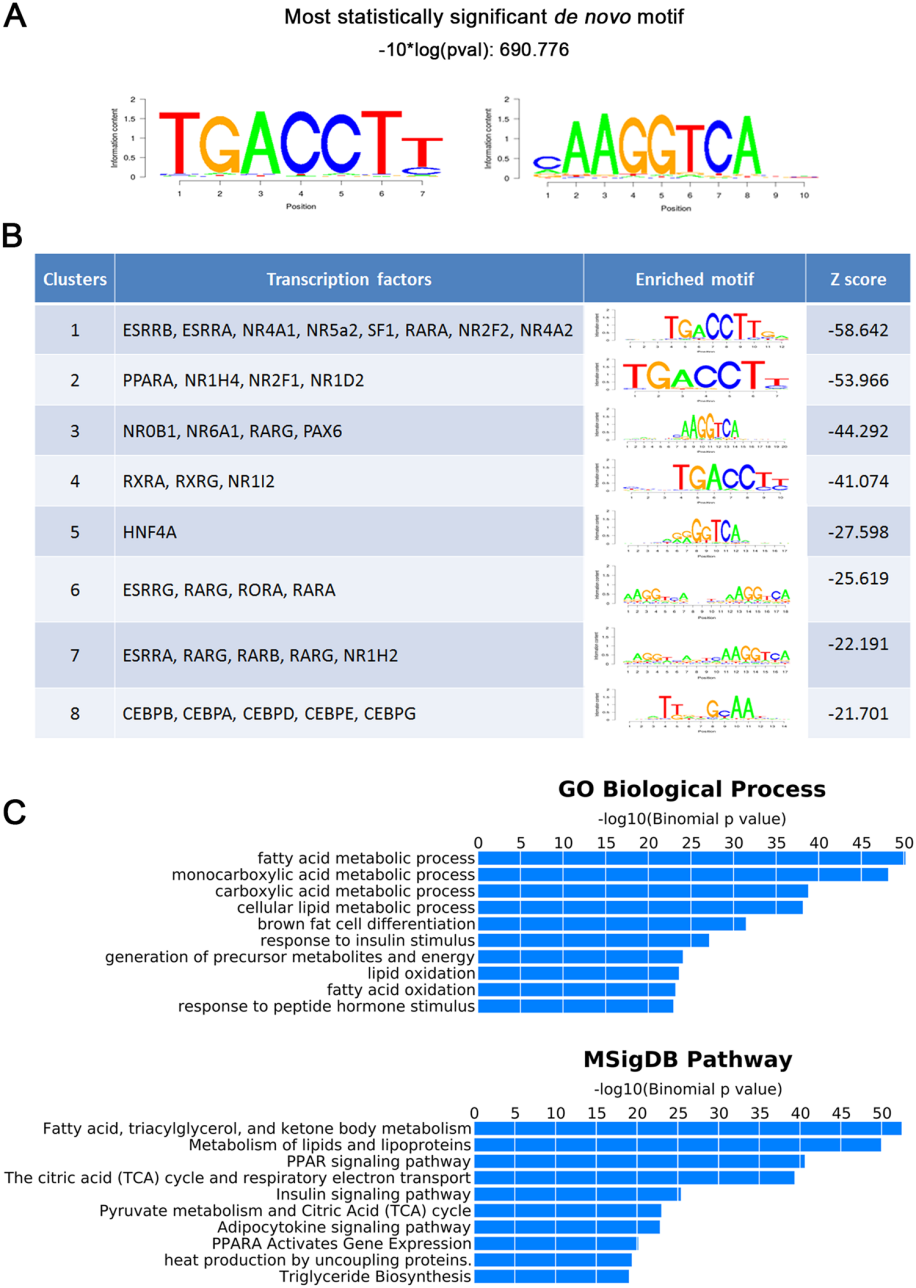
Functional annotation of PGC-1α/NT-PGC-1α ChIP-seq peaks. (**A**) The highest scoring sequence motif compiled from PGC-1α/NT-PGC-1α binding peaks within a 600-bp window centered on the binding summits. (**B**) Transcription factor binding enrichment from the PGC-1α/NT-PGC-1α ChIP-seq. The top eight most enriched motifs identified using the SeqPos motif tool from Galaxy Cistrome. ESRRB (ERR beta), ESRRA (ERR alpha), NR4A1 (NUR77, TR3), NR5A2 (LRH-1, FTF), NR2F2 (TFCOUP2), NR4A2 (NURR1), NR1H4 (FXR), NR2F1 (COUP-TF1), NR1D2 (Rev-Erb), NR0B1 (DAX1), NR6A1 (RTR), NR1I2 (PXR), ESRRG (ERR gamma), and NR1H2 (LXRB). (**C**) Functional enrichment analysis of PGC-1α/NT-PGC-1α peaks using GREAT. Top ten ranked biological processes (GO biological process) and signaling pathways (MsigDB pathway) of genes associated with PGC-1α and NT-PGC-1α peaks are shown.

A *de novo* motif search within the PGC-1α/NT-PGC-1α binding sites revealed the greatest enrichment for the sequences TGACCTT and AAGGTCA that correspond to a NR half-site closely resembling the consensus motif of estrogen-related receptors (ESRRs) (Fig. 3A). ESRRs are key nuclear receptors that function with PGC-1α and NT-PGC-1α to regulate the expression of genes involved in mitochondrial biogenesis, fatty acid oxidation and oxidative phosphorylation^29^. When queried for enrichment of known transcription factor binding motifs, PGC-1α/NT-PGC-1α binding sites had significant enrichment for the consensus motifs of ESRRs, PPARs, RXRs, RARs and HNF4α that are known to cooperate with PGC-1α and NT-PGC-1α (Fig. 3B). Another enriched motif was the consensus motif for CCAAT/enhancer-binding proteins (CEBPs). A previous study has shown that PGC-1α binds to the CEBPβ binding motif^30^, although functional cooperation between CEBPβ and PGC-1α/NT-PGC-1α has not been determined. Taken together, the unbiased identification of these motifs supports the validity of our ChIP-seq data set.

Functional annotation of PGC-1α/NT-PGC-1α binding sites using the Genomic Regions Enrichment of Annotations Tool (GREAT)^31^ further revealed that PGC-1α/NT-PGC-1α-occupied genes were clustered with a range of gene ontology (GO) biological processes including fatty acid metabolism, carboxylic acid metabolism, lipid metabolism, brown adipocyte differentiation and insulin signaling (Fig. 3C, top panel). MSigDB pathway analysis identified significant enrichment with fatty acid and lipid metabolism, TCA cycle, electron transport system, insulin signaling, PPAR signaling, adipocytokine signaling, and heat production (Fig. 3C, bottom panel). Our ChIP-seq results are consistent with the known functions of PGC-1α and NT-PGC-1α in BAT.

### Differential gene expression profile of BAT transcriptome in response to cold

**Figure 4:**
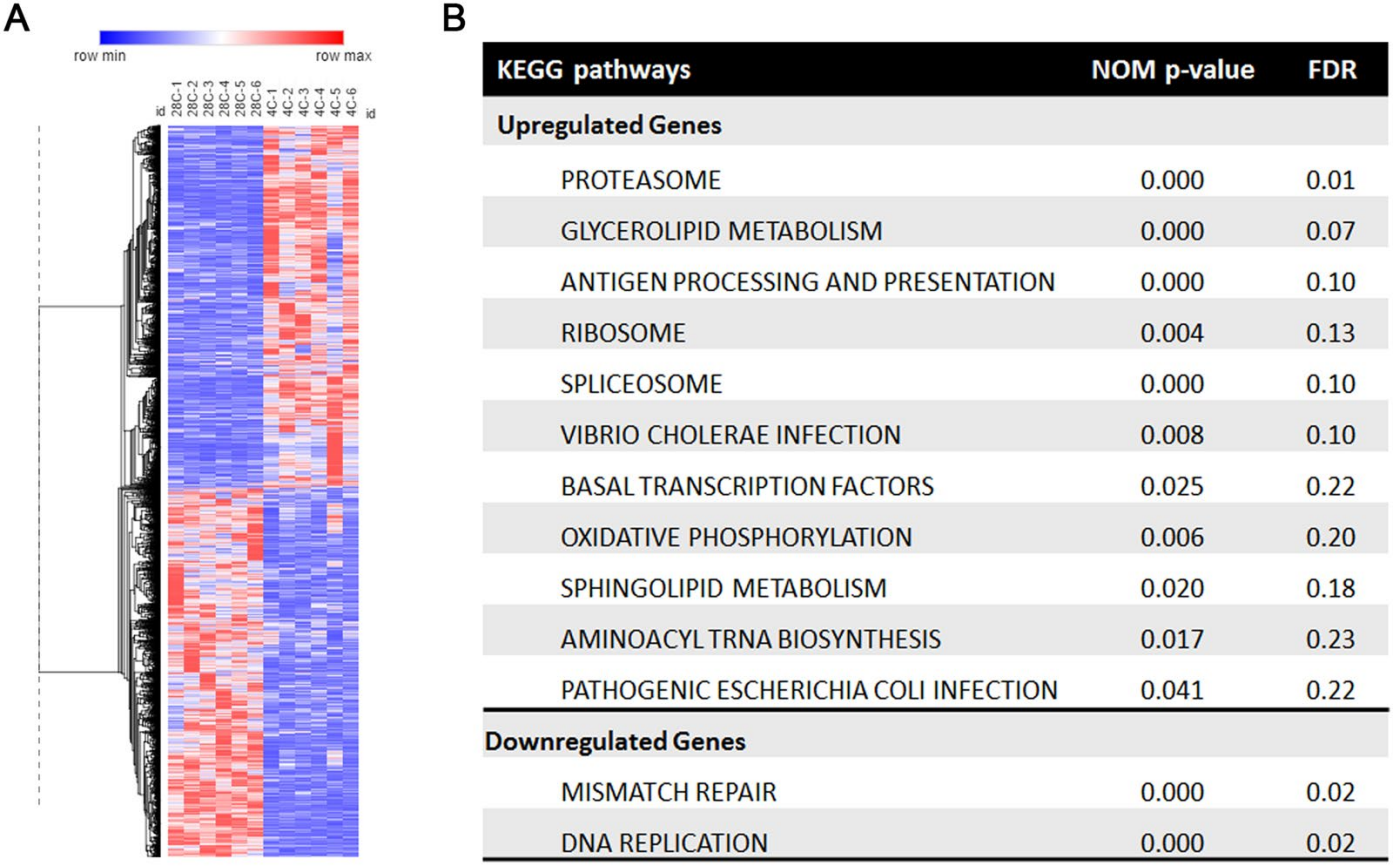
Differential regulation of brown adipose tissue transcriptome by cold. (**A**) A heat map of brown adipose tissue transcriptome that is differentially regulated by cold. Upregulated genes are shown in red and downregulated genes are shown in blue, with gradient colors representing the level of expression (deeper shades for higher degree of differential expression). (**B**) Gene set enrichment analysis (GSEA) showing the pathways enriched in upregulated and downregulated genes in response to cold.

Because transcriptional coactivator/transcription factor binding does not necessarily result in transcription of nearby genes, we wanted to determine how many PGC-1α/NT-PGC-1α-occupied genes are transcriptionally regulated in BAT in response to cold. As described in Fig. 1C, we thus performed genome-wide gene expression analysis using BAT samples collected from mice housed at 28 °C or exposed to 4 °C for 6 h. Cold significantly changed the transcriptome in BAT, of which 4680 genes were differentially expressed between 28 °C- and 4 °C-exposed groups with a *P* value cutoff of 0.05 and a relative fold change cutoff of ±1.5 (Fig. 4A). Pathway enrichment analysis was performed on the full gene list using Gene Set Enrichment Analysis (GSEA)^32^. KEGG pathway databases identified significant enrichment with proteasome, glycerolipid metabolism, antigen processing and presentation, ribosome, spliceosome, basal transcription factors and oxidative phosphorylation pathways (Fig. 4B). The majority of genes in these pathways were upregulated. In contrast, cold-responsive genes associated with mismatch repair and DNA replication tended to be downregulated (Fig. 4B).

### Novel and expanded functional roles for PGC-1α and NT-PGC-1α in BAT

**Figure 5:**
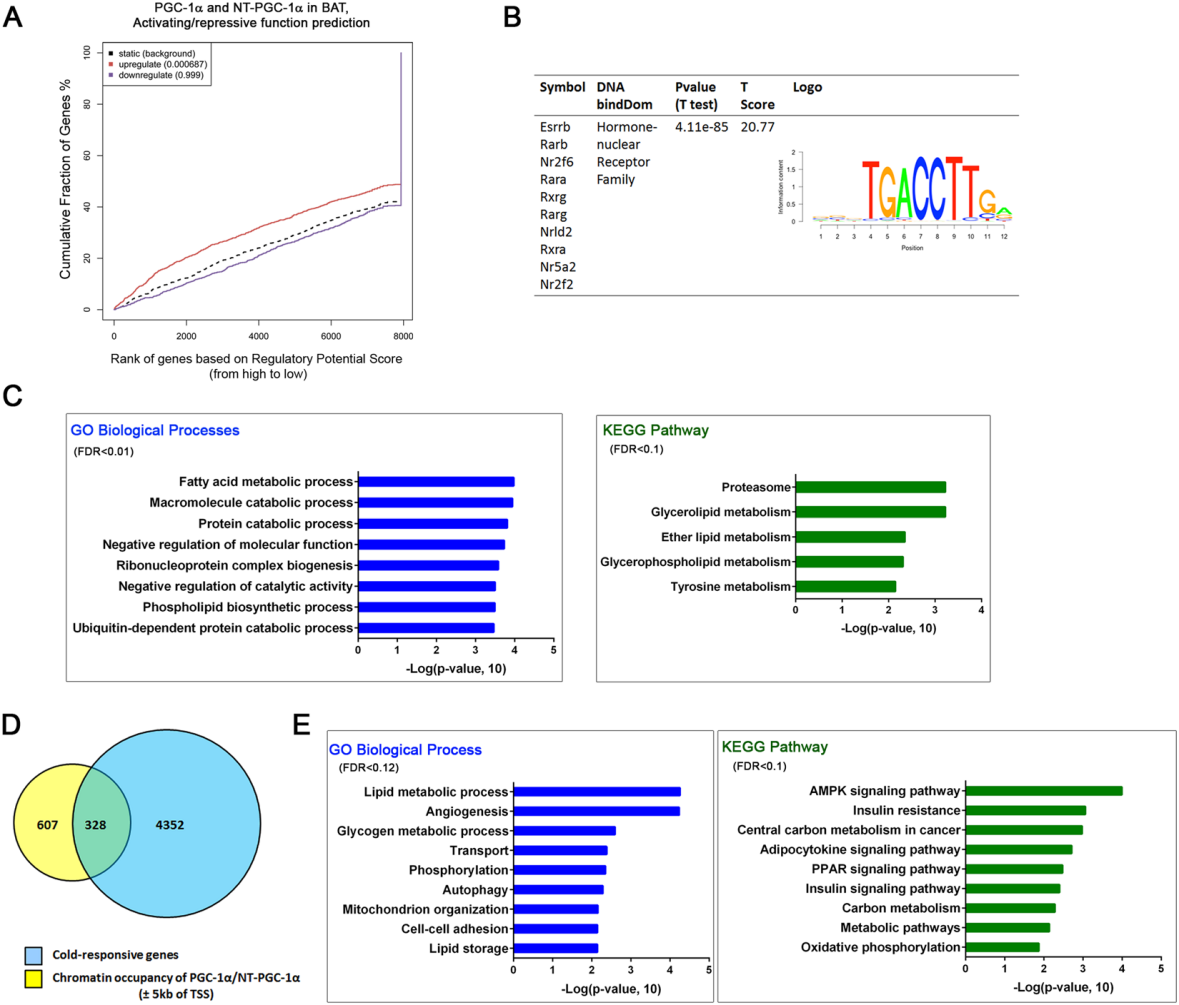
Combined analyses of ChIP-seq and gene expression data. (**A**) Activating and repressive function prediction of PGC-1α and NT-PGC-1α in BAT. BETA software was used with default parameters (peaks within ±100 kb of TSS) to integrate PGC-1α/NT-PGC-1α binding sites with cold-responsive gene expression profiles. The red and purple lines represent putative PGC-1α/NT-PGC-1α-upregulated and -downregulated genes, respectively. Genes are accumulated by the rank according to the regulatory potential score from high to low. *P* values indicate the significance of the activated or repressed group distributions compared with the non-differentially expressed group. (**B**) Binding motif analysis on PGC-1α/NT-PGC-1α-activated target genes. (**C**) Functional GO and KEGG enrichment analysis of PGC-1α/NT-PGC-1α-activated target genes using DAVID. (**D**) Venn diagram showing overlap of PGC-1α/NT-PGC-1α peaks within ±5 kb of TSS with cold-responsive genes in BAT. (**E**) Functional GO and KEGG enrichment analysis of PGC-1α/NT-PGC-1α-activated target genes with peaks within ±5 kb of TSS.

We next performed a Binding and Expression Target Analysis (BETA)^33^ with default parameters (peaks within ±100 kb of TSS) to assess the association of PGC-1α/NT-PGC-1α binding sites with gene expression changes in response to cold. Figure 5A shows cumulative faction of genes ranked by the regulatory potential score from high to low. Supporting that PGC-1α and NT-PGC-1α function as transcriptional coactivators, PGC-1α/NT-PGC-1α binding sites were highly associated with cold-activated genes (Fig. 5A). Motif analysis further revealed that the PGC-1α/NT-PGC-1α binding sites associated with cold-activated genes were greatly enriched for the consensus motif corresponding to the hormone-nuclear receptor family including ESRRs (Fig. 5B). To predict which of the putative PGC-1α/NT-PGC-1α direct target genes might contribute to cold-induced changes of BAT function, we used DAVID database to analyze the functional categories associated with the candidate direct target genes. PGC-1α/NT-PGC-1α-activated target genes were enriched for GO biological processes such as fatty acid metabolism, macromolecule catabolism, protein catabolism, ribonucleoprotein complex biogenesis, phospholipid biosynthesis, and ubiquitin-dependent protein catabolic process (Fig. 5C). In addition, KEGG pathway analysis identified significant enrichment with proteasome, glycerolipid metabolism, ether lipid metabolism, glycerophospholipid and tyrosine metabolism pathways (Fig. 5C). These results not only support the known role of PGC-1α and NT-PGC-1α in fatty acid and lipid metabolism but also suggest their novel role in protein catabolism, ribonucleoprotein complex biogenesis, and ubiquitin-proteasome pathway.

Further investigation into a subset of PGC-1α/NT-PGC-1α target genes (328 genes) that have peaks within ±5 kb from the TSS revealed a significant enrichment of these genes in GO biological processes related to lipid metabolism, angiogenesis, glycogen metabolism, transport of nutrient/protein/solute/electron/proton, phosphorylation, autophagy, mitochondrial organization, cell-cell adhesion, and lipid storage (Fig. 5D,E). The list of genes in each pathway can be found as Supplementary Table S1. KEGG pathway analysis identified enrichment of PGC-1α/NT-PGC-1α target genes in AMPK signaling, insulin signaling, central carbon metabolism, adipocytokine signaling, PPAR signaling, and oxidative phosphorylation (Fig. 5D,E). Taken together, these results suggest that PGC-1α and NT-PGC-1α play a more extensive role in diverse biological processes and signaling pathways involved in cold-induced remodeling of BAT.

## Discussion

PGC-1α and NT-PGC-1α are key transcriptional regulators of cold-induced BAT thermogenesis. In response to cold/β-AR stimulation, PGC-1α and NT-PGC-1α activate transcription of many genes involved in mitochondrial biogenesis, fatty acid oxidation, respiration, and thermogenesis. In this study, we have utilized an unbiased genomic approach to identify the complete repertoire of PGC-1α and NT-PGC-1α target genes in BAT. We mapped PGC-1α/NT-PGC-1α binding sites across the mouse genome in cold-activated BAT (ChIP-seq) and assessed gene expression profile (SAGE-seq) in response to cold. Functional categories enriched in both data sets revealed that PGC-1α and NT-PGC-1α binding sites were highly associated with cold-activated genes and that PGC-1α and NT-PGC-1α affected diverse biological processes associated with fatty acid metabolism, macromolecule catabolism, and protein catabolism, ribonucleoprotein complex biogenesis, phospholipid biosynthesis, and ubiquitin-dependent protein catabolic process. These findings not only support the known roles of PGC-1α and NT-PGC-1α in fatty acid and lipid metabolism but also suggest their novel roles in protein catabolism, ribonucleoprotein complex biogenesis, and ubiquitin-proteasome pathway. Many PGC-1α/NT-PGC-1α binding sites were located at the proximal and/or distal regions of the TSS, indicating that PGC-1α and NT-PGC-1α regulate transcription of these genes by binding to promoter and/or enhancer regions. Moreover, PGC-1α/NT-PGC-1α binding sites contained the consensus motifs corresponding to nuclear receptors (ESRRs, PPARs, RXRs and RARs), suggesting that PGC-1α and NT-PGC-1α cooperate with these nuclear receptors at promoter and/or enhancer regions to activate expression of genes involved in the above mentioned biological processes. However, our results do not provide deeper insight into the relative contribution of PGC-1α and NT-PGC-1α to their target gene expression in BAT since the PGC-1α antibody used for ChIP experiments did not allow to discriminate between the two isoforms. We recently identified an isoform-specific role for NT-PGC-1α in the regulation of mitochondrial DNA transcription^34^. However, our ChIP-seq had a limitation to analyze our previous finding because ChIP samples were prepared from nuclear extracts of BAT.

Investigation into a subset of target genes in which PGC-1α/NT-PGC-1α binding sites are located within ±5 kb of the TSS revealed a number of genes involved in lipid metabolism, angiogenesis, glycogen metabolism, transport of nutrient/protein/solute/electron/proton, phosphorylation, autophagy, mitochondrial organization, and fat storage. These results are in agreement with previous findings that one or more genes involved in these pathways are regulated by PGC-1α and/or NT-PGC-1α at their promoter regions^17–19,23,35–37^. Although 75% of PGC-1α/NT-PGC-1α target genes identified (Table S1) were upregulated by cold, 25% of genes were downregulated. This downregulation may be explained in part by previous findings that transcriptional repressors (e.g. RIP140 and Bhlhe40) co-occupy PGC-1α-targeted gene promoters/enhancers and suppress PGC-1α activity^38,39^.

Collectively, our integrated data sets not only validate the role of PGC-1α and NT-PGC-1α in BAT but also expand the repertoire of PGC-1α and NT-PGC-1α functional target genes. Moreover, identification of PGC-1α/NT-PGC-1α binding sites across the genome and their putative regulatory transcription factors establishes the foundation for further investigation into how PGC-1α and NT-PGC-1α regulate gene expression programs in BAT in response to cold.

## Methods

### Mice

The animal experimental protocol for the study was approved by the Institutional Animal Care and Use Committee at the Pennington Biomedical Research Center, and the procedures were carried out in accordance with the approved guidelines. All mice were housed on a 12 h light/12 h dark cycle. For a cold exposure experiment, 11 to 12-week-old C57BL/6 J male mice were acclimated at 28 °C for 4 days and exposed to 4 °C for 6 h. Mice acclimated at 28 °C for 4 days were used as a control group.

### Western blot and Immunoprecipitation

Brown adipose tissues were lysed in RIPA buffer^40^ and subjected to Western blot analysis using monoclonal anti-PGC-1α^20^ and anti-β-actin antibodies (Sigma). For immuno-precipitation, the protein lysates were incubated with IgG or polyclonal anti-PGC-1α^20,22^ overnight at 4 °C, followed by incubation with protein A agarose beads for 3 h at 4 °C. After washings, immunoprecipitates were subjected to Western blot analysis. Protein concentration was determined using Bio-Rad DC protein assay reagents according to the manufacturer’s instructions.

### ChIP assays

Chromatin immunoprecipitation experiments were performed using interscapular brown adipose tissues (BAT) extracted from mice exposed to 4 °C for 6 h. Briefly, BAT tissues were chopped into small pieces, disaggregated by passing through a 20 G needle, and fixed with formaldehyde. After incubation for 10 min, 2.5 M glycine was added to a final concentration of 0.125 M and the material was pelleted by centrifugation at 8,000 rpm and resuspended in ChIP lysis buffer (10 mM Tris-HCl, pH 8.0, 10 mM NaCl, 3 mM MgCl_2_, 0.5% (vol/vol) NP-40, protease inhibitors). The homogenates were centrifuged at 1,200 × g and the nuclear pellets were resuspended in ChIP shearing buffer (0.25% SDS, 10 mM Tris-HCl, pH 8.0, 1 mM EDTA, protease inhibitors) and sonicated in Covaris MicroTubes to obtain the average DNA fragment size to ~200 bp. For each IP, the sheared chromatin was pooled from at least three mice to minimize the effects of biological variability. Two independent IPs were conducted with rabbit polyclonal PGC-1α antibody directed against the N-terminus of PGC-1α^20,22^. DNA-protein complexes were eluted from protein A beads with elution buffer (100 mM NaHCO_3_, 1% SDS) and reverse crosslinked by adding NaCl to a final concentration of 0.2 M and incubating at 65 °C. ChIP and input samples obtained from two independent experiments were submitted for ChIP-seq library construction.

### ChIP-seq data analysis

ChIP-seq libraries were produced using the SOLiD ChIP-Seq Sample Prep Kit according to the manufacturer’s instruction and sequenced using the Life Technologies 5500XL SOLiD system at the Pennington Biomedical Research Center Genomics Core. Sequence reads that passed quality control filtering were mapped to the mouse reference genome (mm10) using Bowtie^41^ (v1.1.2) and the data from two sequencing runs were concatenated. Peak calling were performed using the MACS algorithm^42^ (v1.0.1) by applying default settings and a *P*-value cutoff of 10^−5^ and by comparing with the input control without immunoprecipitation. Peaks with a FDR < 0.05 were incorporated into further analysis. Mapping of ChIP-seq peaks to the nearest transcription start site (TSS) of the mouse reference genome (mm10) and functional annotation of ChIP-seq peaks were obtained using GREAT^31^ (v3.0.0) with the basal plus extension gene association rule, including curated regulatory domains. For visualization of ChIP-seq peaks on the gene promoters, the Integrative Genomics Viewer (IGV)^43,44^ (v2.3) was used. The web-based application Cistrome Integrative Analysis Pipeline^45^ (http://cistrome.org/ap/) was employed for following analyses: The *cis*-regulatory element annotation system (CEAS) tool used for genomic distribution analysis of ChIP-seq peaks; the SitePro tool used for signal profiling near given peaks; To identify the consensus motifs that are enriched closed to the ChIP-seq peak centers, *de novo* and motif enrichment analyses were performed using the SeqPro motif algorithm with the default parameters (width of region to be scanned, 600 bp; *P*-value < 0.001; mac output hits, 0) and the results were sorted on the basis of z-score. ChIP-seq data are available at the National Center for Biotechnology Information (NCBI)’s Gene Expression Omnibus (GEO) database (accession number GSE110056).

### Analysis of gene expression by SAGE-seq

Interscapular BAT was obtained from mice housed at 28 °C or exposed to 4 °C for 6 h. Total RNA was isolated from six biological replicates per each group using TRI Reagent and RNeasy mini kit. The quantity and quality of purified RNA were determined using an Agilent 2100 Bioanalyzer. Only samples with a RIN number (RNA integrity number) greater than 7.0 were processed further. The SOLiD SAGE technology (Serial Analysis of Gene Expression) was utilized to determine mRNA expression levels by generating the SOLiD SAGE libraries of unique 27-bp sequence tags for all mRNAs and sequencing unique sequence tags isolated from the 3′ ends of mRNAs using the Life Technologies 5500XL SOLiD system. The alignment of sequencing reads to a reference genome was performed using a modified version of the Applied Biosystems SOLiD™ SAGE™ Analysis Software v1.10. Raw count files were analyzed by the R/Bioconductor program DESeq^46^; significance and predictive analyses were performed using SAM (http://www-stat.stanford.edu/~tibs/SAM/index.html) and PAM, respectively^47,48^. The mean values of six biological replicates were determined to identify differentially expressed genes between 28 °C and 4 °C-exposed groups with a *P* value cutoff of 0.05 and a relative fold change cutoff of ±1.5. Unsupervised hierarchical clustering and heat maps were generated using the Morpheus^49^. Pathway enrichment analysis was performed using Gene Set Enrichment Analysis (GSEA)^32^. Gene expression data have been deposited in the Gene Expression Omnibus (GEO) database with accession number GSE110056.

### Integrative analysis of ChIP-seq and expression data

The combined analysis of our ChIP-seq and transcriptomic data was performed using the Binding and Expression Target Analysis (BETA) software available as part of the Cistrome Integrative Analysis Pipeline^33^ (http://cistrome.org/BETA/). The BETA with default settings was used to predict the active or repressive function of PGC-1α/NT-PGC-1α, to identify the candidate target genes that are transcriptionally regulated by PGC-1α/NT-PGC-1α in response to cold, and to identify PGC-1α/NT-PGC-1α binding motifs that are associated with candidate target regions. PGC-1α/NT-PGC-1α target genes were screened out and their significantly enriched Gene Ontology (GO) terms and KEGG pathways were obtained using the web-based DAVID Bioinformatics Resources database^50,51^. A *P* value cutoff of 0.05 was used to identify significantly enriched categories.

## Electronic supplementary material

Supplementary Information
